# German Review

**Published:** 1891-06

**Authors:** Leonard Pearson


### SELECTIONS FROM FOREIGN JOURNALS.

### *GERMAN.*

By LEONARD PEARSON, B. S., V. M. D.

### SODIUM CHLORIDE AS A THERAPEUTIC AGENT.
Vet. Imminger—Donauwoerth.

I. recommends tracheal injections of 50 c.cm. of a 5 per cent. Na Cl solution twice daily, in the horse and cow, to combat weak heart, connected with anæmia, or the result of hemorrhage. A beneficial action can, of course. only be expected when the red blood corpuscles are not too much diminished in number. If disease of the respiratory tract does not permit of a tracheal injection, the Na Cl solution is introduced per rectum. The rectum is first freed from fæces by injecting 10–20 gr. of glycerine, and then 4 to 6 liters of a 3 to 5 per cent Na Cl solution are injected; this is applied twice daily, and continued until an improvement in the condition of the animal sets in.

In cases of luxation of the patella 5 to 10 c.cm., or even more, of a 15 to 20 per cent. solution of Na Cl are injected above the region of the patella. The injection generally causes considerable pain and extensive swelling of the surrounding tissues. As the result of the swelling the animal moves the limb, to a very little extent, and to this circumstance Donauwoerth ascribes much of the beneficial action of the salt injection.

For the treatment of bursal enlargements of the ankle joint 15 to 30 c.cm. are to be injected on each side of the joint; the needle should not be plunged too deeply into the tissues, but directed between the connective tissue and the skin. To prevent the formation of an abscess, care should be taken that the skin and hair, as well as the needle and syringe be thoroughly disinfected; for the disinfection of the skin use a one per cent. sublimate soap.

In inguinal hernia he achieved good results by injecting 5 c.cm. of Na Cl solution near the anterior border of the rupture and the same quantity near the posterior border; care should be taken that the intestine does not become united to the wall of the abdomen during the inflammatory process. *Wochenschrift für Thierheilkunde, No, 6, 7, 8.*

---

### Fluid Extract of Hydrastis Canadensis as a Remedy for Purpura Haemorrhagica.

Dr. Lemke, Berlin.

In 1888, Prof. Hutyra, of Budapest, experimented with Ext. Hydrst. canad., fl., in cases of purpura hæmorrhagica and claimed good results from its use. The drug causes contraction of the blood vessels, thus preventing transudation and hemorrhages.

Dr. Lemke has used it in three cases in which he administered, subcutaneously, a daily dose of 5 grains dissolved in 10 grains of water. In two of these cases improvement commenced at once; the swellings on the legs, breast and head grew rapidly smaller, and the hemorrhagic spots in the nose decreased in size and intensity of color. Both horses made full recoveries. The condition of the third horse improved for three days; then diarhœa set in and, in spite of all treatment, increased in severity at such a rapid rate that the horse died. Dr. Lemke is encouraged, by his limited experience, to think that the drug in question has a beneficial effect on this disease and recommend further trials.

It is necessary, in order to avoid inflammation at the point of injection, to have the syringe well sterilized and to use the purest materials in making the solution. *Zeitschrift fur Veterinarkunde, for May, 1891.*

---

### Echinococcus Larvae in the Thorax of a Horse.

Lienaux (Anales méd. vét. de Brux. Août. 1890.) found a multitude of echinococcus larvæ in the thorax of a horse. These parasites were situated principally on the pleura; the lower part of the right side being entirely covered with cysts varying in size from that of a pea to that of a child's head. They were also found on the left side, on the diaphragm and mediastinum. There were, in some places, adhesions between the pulmonary and costal pleuræ. There were a few cysts in the substance of the lung. Some cysts were also found in the subcutis and a fluctuating swelling between the twelfth and sixteenth ribs was discovered to be an echinoccus cyst that was in direct communication with the cysts within the thorax. All of these parasites belonged to the echinococcus polymorphus. The horse had not been observed to have any respiratory difficulty until near the end of his life. *Zeitschrift fur Veterinarkunde for May, 1891.*

---

### Antiseptic Bandage.

As an antiseptic bandage for parts of the body that can not be readily covered by the ordinary roller or cloth bandages the *Pharmæ Ztg. No. 26* recommends the following mixture: Zinc. oxide., Aqua dest., of each fifty grms; Zinc chlorat., five or six grams. This makes an elastic paste which adheres firmly to the skin, is light and forms a highly satisfactory germ-proof bandage. It is best not to mix the paste until the moment that it is to be used. *Zeit. f. Veterinarkunde, II. 2.*

---

### Camphor.

Camphor has the effect of making iodoform more soluble. For example: ten grams of saturated alcoholic solution of camphor will dissolve one gram of iodoform, while the same amount of pure alcohol will dissolve but 0. 12 grams of iodoform. *Deut. Med. Ztg. No. 22.*

---

### Quittor.

In seven cases of quittor in which the lateral cartilage was excised, in the clinic of the Dresden Vet. School, the horses were able to do hard work, on an average, thirty-one days after the operation was performed. *Wochenschrift für Thierheilkunde, No. 50.*

---

### Bog Spavin.

Veterinarian Zimmer, of Münchenberg, treated two cases of bog spavin for a long time with iodine, blisters, bandages, massage, etc., without result. As a last resort he punctured the joints with a hypodermic needle, drew off the synovia, and injected a one-thousandth solution of corrosive sublimate, which was at once pressed out again and followed by a sharp blister of the entire hock region. The result was a perfect cure in each case. Much stress is to be laid upon the antiseptic manner in which he performed his operations. *Wochenschrift fur Thierheilkunde, No. 44.*

